# Transient spurious intrathecal immunoglobulin synthesis in neurological patients after therapeutic apheresis

**DOI:** 10.1186/s12883-015-0515-x

**Published:** 2015-12-11

**Authors:** Benjamin Berger, Tilman Hottenrott, Jonas Leubner, Rick Dersch, Sebastian Rauer, Oliver Stich, Harald Prüss

**Affiliations:** Department of Neurology and Neurophysiology, University Hospital Freiburg, Breisacher Strasse 64, D-79106 Freiburg, Germany; Department of Neurology and Experimental Neurology, Charité University Medicine Berlin, Charitéplatz 1, D-10117 Berlin, Germany

**Keywords:** Guillain-Barré syndrome, Plasmapheresis, Immunoadsorption, Cerebrospinal fluid, Intrathecal synthesis of immunoglobulins

## Abstract

**Background:**

The analysis of cerebrospinal fluid (CSF) is usually done under steady-state conditions, when proteins (e.g., immunoglobulins) reach diffusion equilibrium between blood and CSF. However, little data has been published on CSF analysis under non-steady-state conditions after therapeutic apheresis. By reducing serum proteins (e.g., immunoglobulins), while leaving CSF unchanged, therapeutic apheresis might cause spuriously altered intrathecal immunoglobulin fractions.

**Methods:**

Based on the incidental finding of plasma exchange-induced increased intrathecal immunoglobulin fractions in a cohort of 12 unsystematically selected patients with various neurological disorders, we retrospectively investigated CSF results that had been raised during routine diagnostic work-up from 41 consecutive neurological patients (predominantly Guillain-Barré syndrome and autoimmune encephalitis) treated with plasmapheresis or immunoadsorption in a tertiary care university hospital in whom lumbar puncture (LP) was performed after a varying number of treatments of therapeutic apheresis.

**Results:**

Only when LP was performed 1 day after therapeutic apheresis, spurious quantitative intrathecal immunoglobulin (Ig) synthesis of at least one subclass (IgG, IgA and/or IgM) was found in 68.4 % of the patients, irrespective of the number of treatments, in all age groups and independent of other previous immunotherapies (e.g., steroids). This phenomenon occurred only transiently and was almost always accompanied by an elevation of the IgG index. In one patient, an elevated IgG index was noticed even 2 days after plasmapheresis. Neither quantitative Ig synthesis, nor elevated IgG index was observed when the LP was performed three or more days after therapeutic apheresis.

**Conclusions:**

Spurious quantitative intrathecal Ig synthesis and increased IgG index are common findings shortly after plasmapheresis or immunoadsorption due to altered serum immunoglobulin levels. Knowledge of this phenomenon is needed for clinicians to prevent false interpretations leading to unnecessary diagnostic and therapeutic procedures. Misdiagnoses can be avoided by considering the characteristic CSF constellation including absence of oligoclonal bands and the close temporal relation to therapeutic apheresis.

## Background

The selective and dynamic function of the blood-cerebrospinal fluid (CSF) barrier and the diffusion of blood-derived proteins (for example, immunoglobulins) into CSF has been described by Reiber et al. [1, 2]. The results of simultaneous measurements of blood-derived proteins in CSF and serum are usually expressed quantitatively as a CSF/serum quotient (e.g., immunoglobulin G quotient Q_IgG_). In some inflammatory neurological disorders of infectious or autoimmune origin (e.g., neuroborreliosis, other viral and bacterial infections, and multiple sclerosis), the disturbance of immunoglobulin (Ig) and albumin quotients leads to an increase of the quantitatively calculated intrathecal Ig fractions indicative of an intrathecal antibody production by local B-cells. In this regard, intrathecal Ig synthesis strongly suggests a neuroinflammatory process [3].

In various neuroimmunological disorders (e.g., Guillain-Barré syndrome, autoimmune encephalitis), plasmapheresis (plasma exchange, PE) or immunoadsorption (IADS), taken together as therapeutic apheresis, are the treatments of choice [4]. CSF analysis plays a key role in the diagnosis of these disorders and is usually performed before starting treatment [5, 6]. Occasionally, during or shortly after therapeutic apheresis, a follow-up CSF analysis becomes necessary for various reasons (e.g., control of CSF cell count or pathogenic antibodies) in clinical routine.

By a transient and isolated reduction of serum proteins, while leaving CSF relatively unchanged, PE or IADS might cause spuriously altered results in the quantitative CSF analysis. In particular, the relative CSF Ig fractions and the respective IgG index are expected to increase, based on the current mathematical concepts used to describe CSF protein composition and dynamics [3]. This could misleadingly suggest an intrathecal antibody synthesis, and hence prompting unnecessary extra diagnostics for search of an infectious or another autoimmune disease (e.g., multiple sclerosis). Although this phenomenon is acknowledged by CSF experts, systematic data proving the existence of this “spurious quantitative intrathecal synthesis” are not available, and, to our knowledge, there is to date only one case report describing spurious intrathecal Ig synthesis after PE [7].

The objective of the present study was to retrospectively determine intrathecal Ig fractions from patients with various inflammatory neurological disorders (predominantly Guillain-Barré syndrome, and autoimmune encephalitis), and compare these before and after PE or IADS treatment to estimate the influence of therapeutic apheresis.

## Methods

### Patients

Initially, an unsystematically selected cohort of 12 patients from the Department of Neurology at the Charité University Medicine Berlin (Germany) receiving LP immediately after treatment with PE between 2013 and 2014 (the Berlin study cohort) was investigated for the occurrence of spurious quantitative intrathecal immunoglobulin synthesis. To confirm the findings, we systematically investigated this phenomenon in a second, independent patient cohort at the Department of Neurology at the University Medical Center Freiburg, Germany (the Freiburg study group). The following inclusion and exclusion criteria were applied to avoid selection bias:

#### Inclusion criteria

At the University Medical Center Freiburg (Germany), electronic databases were screened for neurological patients who were treated with either plasmapheresis (PE) or immunoadsorption (IADS) between 2005 and 2014. Further inclusion criteria required subjects to be aged ≥ 18 years and to have had at least one LP (including a CSF and serum sample taken on the same date) done within two months after PE or IADS.

#### Exclusion criteria

We excluded all patients with neurological diseases (e.g., patients with multiple sclerosis or autoimmune encephalitis), which commonly cause intrathecal Ig synthesis. Exceptions were patients with these conditions who had no intrathecal Ig synthesis in a CSF analysis before or more than 2 months after therapeutic apheresis. Patients with hemolytic CSF samples were excluded from further analysis. Because our main focus in further analyses was on intrathecal Ig fractions, we excluded patients with incomplete CSF and serum data, particularly those lacking Ig analysis.

All patients gave written consent for LP during clinical routine diagnostics. The ethics committee of the University Medical Center Freiburg approved the study.

### Demographic and clinical data

Data regarding demographics (age, gender), neurological diagnoses resulting in treatment with PE or IADS, dates and number of treatments of therapeutic apheresis, and other immunotherapies applied within 4 weeks before these interventions were collected from electronic medical records.

### CSF analysis

In some patients LP was performed within, in others after a complete apheresis cycle consisting of up to six treatments. The methodology of CSF and serum analysis was equivalent in both centers and according to the German Society for Cerebrospinal Fluid Diagnostics and Clinical Neurochemistry. The following routine laboratory CSF data were collected from medical records: time interval between the last treatment of PE or IADS and LP; total protein concentration; albumin concentration in CSF and serum; albumin quotient; immunoglobulin (G, M, and A) concentrations in CSF and in serum; immunoglobulin (G, M, and A) quotients, as well as a quantitative analysis of intrathecal immunoglobulin (G, M, and A) fractions (IF; %) according to the formula Ig_IF_ = [1-Q_Lim_(Ig)/Q_Ig_] × 100 [%] established by Reiber et al. [2]; IgG index [8]; and finally, the presence of oligoclonal bands (OCBs) in CSF and in serum. Concentrations of CSF and serum proteins were measured nephelometrically (ProSpect System, Dade Behring, Siemens, Germany) according to the manufacturer’s instructions. For detection of OCBs, CSF and serum samples were diluted to IgG concentrations of 20 mg/l followed by isoelectric focusing (IEF; Hydragel 9 CSF Isofocusing, Sebia, France) and immunofixation. Intrathecal Ig synthesis was considered significant if IF-values exceeded 10 % in the quantitative analysis. An elevated IgG index was defined as ≥ 0.65 and/or an increase of > 30 % compared to values of an earlier LP. The term “spurious quantitative intrathecal synthesis” is used in the context of this study if elevated intrathecal immunoglobulin fractions or an increased IgG index were caused by the non-steady-state condition between serum and CSF occurring shortly after therapeutic apheresis.

### Statistical analysis

Because of the unsystematic patient selection of the Berlin study cohort we did not pool data with the Freiburg study group. The two-tailed Mann–Whitney *U* test was used for statistical analyses comparing CSF results before and after therapeutic apheresis within the *Freiburg study group*. A *p*-value of < 0.05 was regarded as statistically significant.

## Results

### The Berlin study cohort

Of the 12 patients treated between 2013 and 2014, ten were females, and two were males. The average age was 46 years (range 22–77 years). Eight patients were diagnosed with autoimmune encephalitis, two with neuromyelitis optica, one with multiple sclerosis, and one with myelitis. In these 12 patients, between five and ten treatments of PE were conducted. Six of the 12 patients had an LP 1 day after PE that showed increased quantitative intrathecal immunoglobulin fractions (one of all three classes, two of IgG and IgA, and three of IgG), supporting the hypothesis of spurious intrathecal Ig synthesis. By contrast, the remaining six patients who had an LP more than 1 day after PE with a median time interval to CSF analysis of 18.0 days (range 3–26 days) did not demonstrate increased intrathecal immunoglobulin fractions. Of the six patients with spurious intrathecal Ig synthesis, one patient with autoimmune encephalitis and IgG as well as IgA synthesis had persistence of an increased IgA fraction in the further course, whereas the other five (two with autoimmune encephalitis, two with neuromyelitis optica, and one with myelitis) had a CSF analysis before PE without increased intrathecal immunoglobulin fractions. In these six patients LP showing increased intrathecal Ig fractions was performed after a median number of three apheresis treatments (range 1–6 treatments).

### The Freiburg study group

At University Medical Center Freiburg, 346 neurological patients who were treated with either PE or IADS between 2005 and 2014 were identified. The vast majority (234 cases) had to be excluded since no CSF analysis had been performed within two months after therapeutic apheresis; an additional 50 had incomplete CSF data (e.g., no serum taken). In addition, eight had neurological disorders with intrinsic intrathecal Ig synthesis (e.g., multiple sclerosis) and had no other CSF analysis showing the absence of quantitative intrathecal Ig synthesis. In 13 patients, data on therapeutic apheresis were not available. Finally, 41 patients fulfilled all study criteria.

**Fig. 1:**
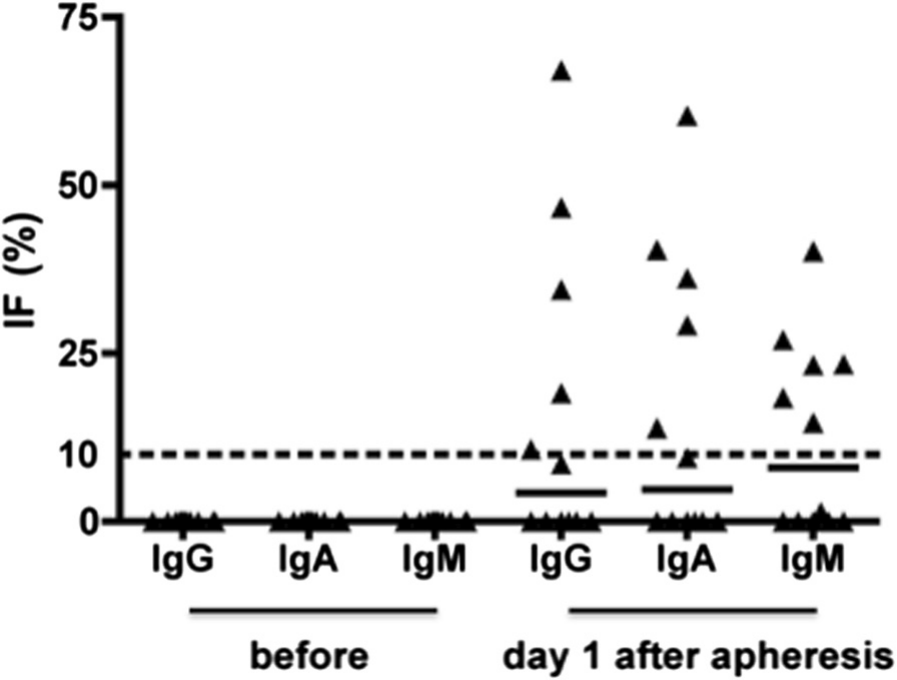
Scatter plot showing the percentage of intrathecal immunoglobulin fractions in patients from the *Freiburg study group* of the IgG, IgA, and IgM class before (left three columns) and 1 day after (right three columns) therapeutic apheresis. Only patients with available, complete CSF and serum data before and 1 day after therapeutic apheresis were included in this graph (*n* = 12). Each triangle represents one patient; the line within the triangles of each column represents the median. Intrathecal immunoglobulin fractions were considered relevant if they exceeded 10 % (as indicated by the dotted line). Abbreviations: IgG, IgA, and IgM = immunoglobulins G, A, and M; IF = intrathecal fraction

The mean age at the time of therapeutic apheresis was 58 years (range 24–80 years); gender distribution was balanced (21 females and 20 males; Table 1). The majority (63.4 %) was diagnosed with Guillain-Barré syndrome (other diagnoses are listed in Table 1). Relevant routine laboratory CSF results of all 41 patients who underwent 84 consecutive LPs are listed in Table 2. Thirty-four of them had an additional LP before and nine had a further follow-up LP. Thirty-seven patients were treated with PE, and four were treated with IADS. In 19 patients (46.3 %), LPs were performed 1 day after therapeutic apheresis, with the number of treatments ranging between one and five. Thirteen patients (68.4 %) showed elevated intrathecal Ig fractions with similar frequencies for all three Ig subclasses (Fig. 1). Of these 13 patients nine had Guillain-Barré syndrome and one chronic relapsing inflammatory optic neuropathy (CRION), multiple sclerosis, paraneoplastic cerebellar degeneration and neuromyelitis optica each. A median number of four apheresis treatments (range 1–5 treatments) were performed before increased intrathecal Ig fractions were detected. Elevated intrathecal Ig fractions were seen in 12 of 37 patients treated with PE and in one of four patients after IADS. In 10 of the 13 patients with increased intrathecal immunoglobulin fractions 1 day after therapeutic apheresis, data from an additional CSF analysis before or during further follow-up, showed no increased fractions. In two patients we had both, showing disappearance of the elevated immunoglobulin fractions during the further course. In one patient with Guillain-Barré syndrome and increased fractions 1 day after PE we had no data of follow-up CSF analysis. Table 3 showing CSF and serum data of a representative patient before and 1 day after PE, demonstrates that reduced serum Ig levels were accompanied by hardly altered CSF results, therefore leading to an increase of the respective quotients and intrathecal immunoglobulin fractions.

**Table 1:** Diagnoses of the 41 patients of the *Freiburg study group*

Diagnosis	
Guillain-Barré syndrome, n (%)	26 (63.4)
Autoimmune encephalitis, n (%)	6 (14.6)
Miller-Fisher syndrome, n (%)	2 (4.9)
Chronic relapsing inflammatory optic neuropathy (CRION), n (%)	1 (2.4)
Multiple sclerosis, n (%)	1 (2.4)
Parainfectious myelitis, n (%)	1 (2.4)
Myasthenia gravis, n (%)	1 (2.4)
Anti-Yo-associated paraneoplastic cerebellar degeneration, n (%)	1 (2.4)
Anti-Hu-associated polyneuropathy, n (%)	1 (2.4)
Neuromyelitis optica, n (%)	1 (2.4)

**Table 2:** CSF parameters of 41 patients undergoing therapeutic apheresis *(Freiburg study group)*

CSF analysis				
	Prior to treatment	One day after treatment	Two days after treatment	More than two days after treatment (median 14.0 days; range 3–55; SD 13.5)
n (%)	33 (80.5)	19 (46.3)	4 (9.8)	18 (43.9)
Intrathecal IgG synthesis, n (%)	0 (0.0)	9 (47.4)	0 (0.0)	0 (0.0)
Intrathecal IgA synthesis, n (%)	1 (3.1)	7 (36.8)	0 (0.0)	0 (0.0)
Intrathecal IgM synthesis, n (%)	1 (3.1)	8 (42.1)	0 (0.0)	0 (0.0)
IgG index ≥ 0.65, n (%)	6 (18.8)	18 (94.7)	1 (25.0)	1 (5.6)
OCBs predominantly in CSF, n (%)	1 (3.1)	0 (0.0)	0 (0.0)	1 (5.6)

Abbreviations: *CSF* cerebrospinal fluid, *IgG, IgA, and IgM* immunoglobulins G, A, and M, *OCBs* oligoclonal bands, *SD* standard deviation

**Table 3:** Results of CSF and serum analyses of a representative study patient

CSF/serum biomarker [normal range]	LP before PE	LP one day after PE
CSF total protein concentration (mg/L) [<450]	451	820
CSF albumin (mg/L)/serum albumin (g/L) [110-350/35-52]	276/41.9	471/53.2
Q_Alb_ [for age 50 years: < 7.3]	6.5	8.9
CSF IgG (mg/L)/serum IgG (g/L) [14-40/7-16]	26.9/9.79	16.7/2.23
_QIgG_	2.7	7.4
_IgG synthesis (%)_	0	8.6
_IgG index_	0.41	0.84
CSF IgA (mg/L)/serum IgA (g/L) [1.5-6.0/0.7-4.0]	4.38/2.3	3.57/0.54
QIgA	1.9	6.6
IgA synthesis (%)	0	29.2
CSF IgM (mg/L)/serum IgM (g/L) [<1/0.4–2.3]	0.63/1.34	0.75/0.19
QIgM	0.4	4.0
IgM synthesis (%)	0	40.2

Abbreviations: *CSF* cerebrospinal fluid, *LP* lumbar puncture, *PE* therapeutic plasma exchange, *QAlb* albumin quotient, *IgG, IgA, and IgM* immunoglobulins G, A, and M, *QIgG* IgG quotient, *QIgA* IgA quotient, *QIgM* IgM quotient

**Fig. 2:**
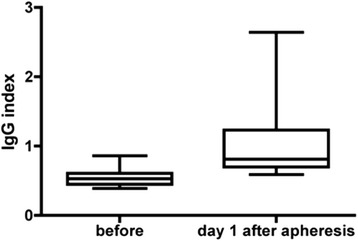
Box-and-whiskers plots showing the IgG index of all patients from the *Freiburg study group* with available, complete CSF and serum data before (baseline, left plot) and 1 day after (day 1, right plot) therapeutic apheresis (*n* = 12). The box plot includes all values from the first (25.0 %) to the third quartiles (75.0 %), while the band inside the box corresponds to the median. The ends of the whiskers represent the minimum and maximum values, respectively. The two-tailed Mann–Whitney test showed a significant difference between the IgG index after, as compared to before, therapeutic apheresis (*p* = 0.0001). Abbreviation: IgG = immunoglobulins G

Elevated intrathecal immunoglobulin fractions were seen after one or more treatments, in patients from all age groups, independent of other previous immunotherapies *(data not shown)*, and in a variety of neurological diagnoses (Table 1). Elevated intrathecal immunoglobulin fractions were always accompanied by an increased IgG index (Fig. 2). Five of the six patients on whom LPs were performed 1 day after therapeutic apheresis, but who had no elevated intrathecal Ig fractions, demonstrated an elevated IgG index (*data not shown*). According to quantitative IF analysis *(data not shown),* none of the 22 patients who underwent LP more than 1 day after therapeutic apheresis showed elevated intrathecal Ig fractions. In one patient, an elevated IgG index was noticed 2 days after PE, but in none of the patients with a CSF analysis three or more days after therapeutic apheresis (*data not shown)*. Strikingly, positive or predominant OCBs in CSF were detected in none of the 19 cases with elevated intrathecal Ig fractions or an elevated IgG index up to 2 days after therapeutic apheresis.

## Discussion

In this study, we describe spurious intrathecal immunoglobulin (Ig) synthesis of all three subclasses (IgG, IgA and IgM) occurring 1 day after therapeutic apheresis in 6 of 12 selected patients (50.0 %, the *Berlin study cohort*), as well as in 13 of 19 patients (68.4 %) from a well-defined, independent, confirmatory cohort of patients with various neurological disorders (the *Freiburg study group*). Additionally, 18 of these 19 patients (94.7 %) had spuriously elevated IgG indexes. While being well-acknowledged among CSF experts, to our knowledge this is the first systematic study on these spurious phenomena.

The relevance of our findings is further illustrated by the fact that the misinterpretation of spurious intrathecal Ig synthesis 1 day after therapeutic apheresis resulted in further specific diagnostics and/or unnecessary treatments in some of our patients. For example, one patient with bilateral facial palsy due to Guillain-Barré syndrome received plasmapheresis as an initial treatment. Since a follow-up LP revealed elevated intrathecal IgM fractions and an ambiguous result for Borrelia burgdorferi serology, treatment with ceftriaxone was started. In another case, elevated intrathecal fractions of all three Ig classes led to an extensive search for infectious pathogens in spite of a normal cell count in CSF analysis.

In our study cohorts spuriously elevated intrathecal Ig fractions were commonly found after therapeutic apheresis only in those cases for which the time interval after therapeutic intervention to CSF analysis did not exceed 1 day. As expected, this finding was not restricted to PE but was also observed after IADS. Furthermore, elevated intrathecal Ig fractions were noticed irrespective of the patient’s age, number of treatments, and other previous immunotherapies. This finding was observed in various neurological disorders, including GBS, autoimmune encephalitis, and multiple sclerosis. A noticeably increased IgG index always accompanied spurious elevated intrathecal Ig fractions and was observed even until 2 days after PE. Due to the “selectivity” of the blood–brain-barrier (BBB) with respect to molecular size [2], one would expect a predominant increase of the intrathecal IgM fraction after therapeutic apheresis. Surprisingly, spurious intrathecal Ig synthesis was found with equal frequency in all Ig classes in this study, including IgG with the smallest molecular size.

The assumption that this phenomenon is spurious is supported by several facts: (1) The *Freiburg study group* mainly consisted of patients with neurological diagnoses typically not associated with intrinsic intrathecal Ig synthesis or elevated IgG index (e.g., Guillain-Barré syndrome) [5]. In cases of diagnoses possibly associated with intrinsic intrathecal Ig synthesis (e.g., multiple sclerosis), these patients were included only if a quantitative CSF analysis before, or long after, therapeutic apheresis did not show intrathecal Ig synthesis. (2) We usually found unaltered Ig CSF concentrations after therapeutic apheresis, while serum Ig concentrations were mostly decreased within the first day after intervention, thereby leading to a relative increase of the respective CSF/serum quotients. A representative patient, whose data is shown in Table 3, illustrates this*.* (3) Elevated intrathecal Ig fractions and elevated IgG index occurred only transiently, i.e., no more than 1 day after PE or IADS for intrathecal Ig fractions and 2 days for IgG index, respectively, and disappeared in the further course, indicating a “steady-state equilibrium” of CSF and serum proteins over time *(data not shown)* [1]. (4) Contrary to the IgG index or to the quantitative analysis of intrathecal Ig fractions using serum/CSF quotients, OCBs measured in Western blots provide qualitative and highly sensitive evidence for intrathecal IgG synthesis and are not susceptible to spurious alterations due to non-steady-state serum/CSF conditions [9]. As stated above, OCBs were detected in none of the 19 cases with elevated intrathecal Ig fractions or with an elevated IgG index up to 2 days after therapeutic apheresis.

To ensure correct interpretation, CSF and serum samples should be analyzed under steady-state conditions allowing proteins (e.g., immunoglobulins) to reach a diffusion equilibrium, since the Reibergrams commonly used for CSF analysis were developed under steady-state serum/CSF conditions [1, 2]. Hence, under non-steady-state conditions (e.g., shortly after PE or IADS), a critical interpretation of CSF results is necessary since a rapid and predominant decrease of immunoglobulins in serum leads to a transient “imbalance” between the two compartments [5].

A limitation of this study is its retrospective character and the small sample size of only 41 systematically selected patients in the *Freiburg study group*. Additionally, we included both patient groups receiving plasmapheresis or immunoadsorption, which are different therapeutic measures [10]. Briefly, therapeutic plasmapheresis removes and replaces the complete plasma, containing among others immunoglobulins, immune complexes and coagulation factors. By contrast, immunoadsorption exclusively removes immunoglobulins and immune complexes from the plasma by a special “absorber”. However, the relevant “pathophysiologic principle” important for this study, namely reducing serum immunoglobulin levels while leaving CSF levels unchanged, is equivalent in both.

## Conclusions

Transiently elevated intrathecal Ig fractions and increased IgG index (“spurious intrathecal immunoglobulin synthesis”), both due to dropped serum Ig levels, are frequent phenomena occurring in the first 2 days following therapeutic apheresis. To avoid unnecessary diagnostic and therapeutic procedures, clinicians should be aware of this spurious phenomenon. Recognition of “spurious intrathecal synthesis” is possible when taking into account the absolute concentrations of CSF and serum Ig. In addition, OCBs should be considered to distinguish between “real” and “spurious” Ig synthesis, as they remain negative in the CSF despite spuriously elevated intrathecal Ig fractions and/or IgG indexes. If CSF analysis of intrathecal Ig synthesis is indicated after therapeutic apheresis, we recommend a minimum time interval of 3 days between apheresis and LP.
